# *β*-Endorphin Mediates the Development and Instability of Atherosclerotic Plaques

**DOI:** 10.1155/2020/4139093

**Published:** 2020-03-28

**Authors:** Taisuke Okano, Kengo Sato, Remina Shirai, Tomomi Seki, Koichiro Shibata, Tomoyuki Yamashita, Ayaka Koide, Hitomi Tezuka, Yusaku Mori, Tsutomu Hirano, Takuya Watanabe

**Affiliations:** ^1^Laboratory of Cardiovascular Medicine, Tokyo University of Pharmacy and Life Sciences, 1432-1 Horinouchi, Hachioji, Tokyo 192-0392, Japan; ^2^Division of Laboratory and Transfusion Medicine, Hokkaido University Hospital, Kita-15 Nishi-7, Kita-ku, Sapporo, Hokkaido 060-8638, Japan; ^3^Division of Diabetes, Metabolism, and Endocrinology, Department of Medicine, Showa University School of Medicine, 1-5-8 Hatanodai, Shinagawa-ku, Tokyo 142-8666, Japan; ^4^Department of Internal Medicine, Ushioda General Hospital/Clinic, Yokohama, Japan

## Abstract

*β*-Endorphin, an endogenous opioid peptide, and its *μ*-opioid receptor are expressed in brain, liver, and peripheral tissues. *β*-Endorphin induces endothelial dysfunction and is related to insulin resistance. We clarified the effects of *β*-endorphin on atherosclerosis. We assessed the effects of *β*-endorphin on the inflammatory response and monocyte adhesion in human umbilical vein endothelial cells (HUVECs), foam cell formation, and the inflammatory phenotype in THP-1 monocyte-derived macrophages, and migration and proliferation of human aortic smooth muscle cells (HASMCs) in vitro. We also assessed the effects of *β*-endorphin on aortic lesions in *Apoe*^−/−^ mice in vivo. The *μ*-opioid receptor (OPRM1) was expressed in THP-1 monocytes, macrophages, HASMCs, HUVECs, and human aortic endothelial cells. *β*-Endorphin significantly increased THP-1 monocyte adhesion to HUVECs and induced upregulation of intercellular adhesion molecule-1, vascular cell adhesion molecule-1, and E-selectin via nuclear factor-*κ*B (NF-*κ*B) and p38 phosphorylation in HUVECs. *β*-Endorphin significantly increased HUVEC proliferation and enhanced oxidized low-density lipoprotein-induced foam cell formation in macrophages. *β*-Endorphin also significantly shifted the macrophage phenotype to proinflammatory M1 rather than anti-inflammatory M2 via NF-*κ*B phosphorylation during monocyte-macrophage differentiation and increased migration and apoptosis in association with c-jun-N-terminal kinase, p38, and NF-*κ*B phosphorylation in HASMCs. Chronic *β*-endorphin infusion into *Apoe*^−/−^ mice significantly aggravated the development of aortic atherosclerotic lesions, with an increase in vascular inflammation and the intraplaque macrophage/smooth muscle cell ratio, an index of plaque instability. Our study provides the first evidence that *β*-endorphin contributes to the acceleration of the progression and instability of atheromatous plaques. Thus, *μ*-opioid receptor antagonists may be useful for the prevention and treatment of atherosclerosis.

## 1. Introduction

Atherosclerosis is a chronic inflammatory response to injury of the arterial wall [1, 2]. Vascular inflammation stimulates the expression of adhesion molecules, such as intercellular adhesion molecule-1 (ICAM-1), vascular cell adhesion molecule-1 (VCAM-1), and E-selectin, in endothelial cells (ECs). These effectors encourage monocyte adhesion and infiltration into the neointima lesion, followed by atheroma formation and subendothelial accumulation of lipid-laden macrophage foam cells [1, 3]. Foam cell formation is characterized by intracytoplasmic accumulation of cholesterol ester and depends on the balance among the uptake of oxidized low-density lipoprotein (oxLDL) via CD36, cholesterol esterification by acyl-CoA:cholesterol acyltransferase-1 (ACAT-1), and the efflux of free cholesterol controlled by the ATP-binding cassette transporter A1 (ABCA1) [2, 3]. Categorization of the macrophage phenotype as either pro- or anti-inflammatory (M1 and M2 phenotypes, respectively) has recently focused on atherosclerosis [2, 4]. In addition, the migration and proliferation of vascular smooth muscle cells (VSMCs) also contribute to the development of atheromatous plaques [1, 2].

*β*-Endorphin, a morphine-like peptide, was identified in brain as an endogenous opioid peptide hormone that is related to pain modulation [5–8]. A 91-residue polypeptide called *β*-lipotropin is biosynthesized from the precursor of adrenocorticotropic hormone pro-opiomelanocortin [9] and is cleaved by trypsin to the 60-amino-acid *β*-melanotropin and 31-amino-acid *β*-endorphin [10]. The human, mouse, and rat *β*-endorphin amino acid sequences are 90% identical [11]. The specific receptor for *β*-endorphin is the *μ*-opioid receptor [8, 12]. *β*-Endorphin is mainly produced and secreted in the pituitary gland [13]. Both *β*-endorphin and *μ*-opioid receptors are expressed in synovial tissue, ECs, monocytes, macrophages, lymphocytes, and granulocytes [14–18]. *β*-Endorphin also ameliorates insulin resistance [8, 19]. This peptide increases endothelin-1 release and decreases nitric oxide release from human ECs and monocytes via *μ*1-opioid receptors, which may lead to endothelial dysfunction [15]. *β*-Endorphin stimulates chemotaxis of monocytes, their differentiation into macrophages, and production of reactive oxygen species, interleukin (IL)-1*β*, IL-10, interferon-*γ*, and tumor necrosis factor-*α* in monocytes/macrophages [20–25]. The *μ*-opioid receptor is upregulated by IL-1*α* and IL-1*β* in ECs [26]. Clinical studies have reported that plasma *β*-endorphin levels are significantly increased in patients with coronary artery disease (CAD) and heart failure [27, 28]. However, the effects of *β*-endorphin on atherosclerosis have not yet been reported.

In the present study, we aimed at clarifying the effects of *β*-endorphin in vitro on the inflammatory response and adhesion of human THP-1 monocytes to human umbilical vein ECs (HUVECs). We also assessed the inflammatory phenotype and foam cell formation in THP-1 monocyte-derived macrophages, as well as migration and proliferation of human aortic smooth muscle cells (HASMCs). Our in vivo studies focused on the development of atherosclerotic lesions in *Apoe*^−/−^ mice.

## 2. Materials and Methods

### 2.1. Materials

THP-1 monocytes and their culture medium RPMI-1640 were purchased from Health Science Research Resources Bank (Osaka, Japan) and Wako (Osaka, Japan), respectively. Human aortic endothelial cells (HAECs), HUVECs, HASMCs, EC growth medium-2 (EGM-2), and smooth muscle cell growth medium-2 (SmGM-2) were purchased from Lonza (Walkersville, MD, USA). Human *β*-endorphin was purchased from Peptide Institute (Osaka, Japan). Platelet-derived growth factor-BB (PDGF-BB) and 2-O-tetradecanoylphorbol-13-acetate (TPA) were purchased from Wako.

### 2.2. Foam Cell Formation Assay

THP-1 monocytes were seeded in 3.5 cm dishes (1 × 10^6^ cells/1 mL/dish). Cells were incubated at 37°C in a 5% CO_2_ humidified incubator for 3 days in RPMI-1640 medium containing 10% fetal bovine serum (FBS), 0.05 mg/mL streptomycin, 50 U/mL penicillin, and the indicated concentrations of *β*-endorphin in the presence of 150 ng/mL TPA to induce differentiation into macrophages. Subsequently, THP-1 monocyte-derived macrophages were incubated for 3 days in the absence of TPA prior to immunoblotting [2, 29–34]. Then, THP-1 monocyte-derived macrophages were further incubated for 2 days in the renewal medium with the same concentrations of *β*-endorphin along with 50 *μ*g/mL oxLDL and 100 *μ*M [^3^H]oleate (PerkinElmer, Yokohama, Japan) conjugated to bovine serum albumin [2, 29–34]. Cellular lipids were extracted, and the radioactivity of cholesterol-[^3^H]oleate was determined with thin-layer chromatography [2, 29–34].

### 2.3. Western Blotting

Aliquots of protein extracts derived from THP-1 monocytes, THP-1-derived macrophages, HASMCs, and HUVECs were separated with 10% sodium dodecyl sulfate-polyacrylamide gel electrophoresis and then immunoblotted with antibodies raised against the following proteins: CD36, CD68, ACAT-1, ICAM-1, VCAM-1 (Santa Cruz Biotechnology, Santa Cruz, CA, USA), ABCA1, phosphorylated nuclear factor-*κ*B (p-NF-*κ*B), phosphorylated c-jun N-terminal kinase (p-JNK), *α*-tubulin, arginase-1 (GeneTex, Irvine, CA, USA), E-selectin, MARCO (Bioss, Woburn, MA, USA), peroxisome proliferator-activated receptor-*γ* (PPAR-*γ*; Signalway Antibody, College Park, MD, USA), phosphorylated Akt, phosphorylated extracellular signal-regulated kinase 1/2 (p-ERK1/2), phosphorylated p38 (p-p38), Bax (Cell Signaling Technology, Danvers, MA, USA), Bcl-2 (Abcam, Cambridge, UK), cleaved caspase-3 (R&D Systems, Minneapolis, MN, USA), glyceraldehyde-3-phosphate dehydrogenase (GAPDH; Acris-OriGene Technologies, Herford, Germany), and *β*-actin (Sigma, St. Louis, MO, USA) [2, 29–40]. Proteins were visualized by enhanced chemiluminescence western blotting detection reagents (GE Healthcare, Amersham, UK).

### 2.4. Conventional Reverse Transcription Polymerase Chain Reaction (RT-PCR)

The mRNA expression of *OPRM1* (*μ*-opioid receptor gene) was determined in THP-1 monocytes, their derived macrophages, HASMCs, HUVECs, and HAECs. Total RNA was extracted using a High Pure RNA Isolation Kit (Roche Diagnostics, Mannheim, Germany). Complementary DNAs were synthesized from isolated RNA templates using a High Capacity cDNA Reverse Transcription Kit (Applied Biosystems, Foster City, CA, USA). The PCR products were visualized with 2% agarose gel electrophoresis [2, 29–40]. The mRNAs for *OPRM1* and *GAPDH* were detected as described previously [2, 29–40].

### 2.5. Quantitative Real-Time RT-PCR

HUVECs at passage 3–7 were seeded in 3.5 cm dishes and incubated at 37°C in a 5% CO_2_ incubator for 24 h in EGM-2. Near-confluent HUVECs were incubated at 37°C in 5% CO_2_ for 4 h with or without the indicated concentration of *β*-endorphin in EGM-2 [34, 35, 38]. Total RNA and cDNA were obtained as described above [34, 35, 38]. qPCR was performed with Power SYBR^®^ Green PCR Master Mix (Applied Biosystems) to quantify mRNA for *ICAM1*, *VCAM1*, *SELE* (E-selectin gene), and *GAPDH*. All reactions were carried out on a StepOnePlus system (Applied Biosystems). Each sample was analyzed in triplicate at least three times for each PCR measurement. Melting curves were checked to ensure specificity. The relative quantification of mRNA expression was calculated using the standard curve method with normalization to *GAPDH*.

### 2.6. Monocyte Adhesion Assay

HUVECs at passage 3–5 were seeded in 24-well plates (0.9 × 10^5^ cells/500 *μ*L/well) and incubated at 37°C in a 5% CO_2_ incubator for 24 h in EGM-2. Confluent HUVECs were incubated for 16 h with 0.5% FBS in EGM-2. Subsequently, cells were incubated for 4 h in 0.5% FBS in EGM-2 containing the indicated concentrations of *β*-endorphin [2, 29–36]. Then, THP-1 monocytes were labeled with CellTrace™ calcein red-orange (Life Technologies, Carlsbad, CA, USA) with a total of 1 × 10^5^ cells added to each well of a HUVEC-seeded 24-well plate. After 1 h, THP-1 monocytes that were bound to HUVECs were washed four times and then examined by fluorescence microscopy (IX70; Olympus, Tokyo, Japan). Their adhesion was assessed using image analysis software (ImageJ; NIH, Bethesda, MD, USA) [2, 29–36].

### 2.7. Migration Assay (Scratch Assay)

HASMCs at passage 6–8 were seeded in 24-well plates (1 × 10^5^ cells/500 *μ*L/well). Cells were grown to near confluence at 37°C in 5% CO_2_ in SmGM-2. HASMCs were then serum-starved overnight in serum-free SmGM-2. To induce a migrating zone in a transverse scratch wound, each monolayer of HASMCs was scratched with a sterilized Cell Scratcher (Iwaki, Tokyo, Japan). HASMCs were gently rinsed to discard debris, initial photomicrographs were taken (0 h), and then cells were incubated for 24 h in serum-free SmGM-2 containing the indicated concentration of *β*-endorphin or PDGF-BB [32]. After overnight incubation (24 h), a second set of images was obtained to measure HASMC migration over the scratched area [32]. The migrating zone was examined and analyzed between 0 h and 24 h using ImageJ software.

### 2.8. Proliferation (Viability) Assay

HUVECs or HASMCs at passage 2–8 were seeded in 96-well plates (1 × 10^4^ cells/100 *μ*L/well) and incubated for 24 h in EGM-2 or SmGM-2. Cells were then incubated for a further 24 h in fresh medium containing the indicated concentration of *β*-endorphin. Ten microliters of WST-8 solution (Cell Count Reagent SF; Nacalai Tesque, Kyoto, Japan) was then added to each well [2, 29–40]. After 1 h of incubation, the quantity of formazan product was determined by reading absorbance at 450 nm using a Sunrise Remote R™ microplate reader (Tecan, Kawasaki, Japan) [2, 29–40].

### 2.9. Apoptosis Assay

HASMCs were seeded in 12-well plates (3 × 10^5^ cells/1 mL/well) and incubated at 37°C in a 5% CO_2_ incubator for 24 h in EGM-2, followed by a 24 h incubation in the same medium containing the indicated concentrations of *β*-endorphin. Cells were fixed with 4% paraformaldehyde in phosphate-buffered saline. Terminal deoxynucleotidyl transferase-mediated deoxyuridine triphosphate-biotin nick end labeling (TUNEL) staining was then performed using an In Situ Apoptosis Detection Kit (Takara Bio, Otsu, Japan) as described previously [2, 29–36]. Nuclei were costained using 6-diamidino-2-phenylindole (DAPI; DOJINDO, Kumamoto, Japan). All samples were then mounted in fluorescence mounting medium (Dako, Glostrup, Denmark) and examined with confocal microscopy (FV1000D, Olympus, Tokyo, Japan). Fluorescence was detected using the excitation wavelengths of 488 nm (TUNEL) and 360 nm (DAPI) [2, 29–36]. The number of TUNEL-positive cells was counted in three fields of view chosen randomly from each sample.

### 2.10. Animal Experiments

Animal experiments were performed in accordance with the National Institutes of Health Guidelines for the Care and Use of Laboratory Animals and were approved by the Institutional Animal Care and Use Committee of Tokyo University of Pharmacy and Life Sciences (no. L19-16). A total of 21 male spontaneously hyperlipidemic *Apoe*^−/−^ mice (KOR/StmSlc-*Apoe*^*shl*^ mice) were purchased from Japan SLC (Hamamatsu, Japan). Mice were fed a high cholesterol diet containing 1.25% cholesterol, 3.0% lard, and 1.625% glucose (F2HFD1, Oriental Yeast, Tokyo, Japan), starting at 13 weeks of age [2, 29–37, 39, 40]. At 13 weeks of age, five mice were sacrificed as preinfusion controls. The remaining 16 were divided into two groups of eight each and were infused with saline (vehicle) or *β*-endorphin (5 *μ*g/kg/h) using osmotic minipumps (Alzet Model 1002; Durect, Cupertino, CA, USA) for 4 weeks. Doses of *β*-endorphin were selected based on others' previous data and our preliminary data [32–35]. Once every 2 weeks, the minipumps were implanted subcutaneously into the dorsum under medetomidine-midazolam-butorphanol anesthesia (0.3 mg/kg, 4.0 mg/kg, and 5.0 mg/kg, respectively, intraperitoneal injection).

### 2.11. Animal Measurements

Four weeks after commencing infusions into *Apoe*^−/−^ mice, systolic and diastolic blood pressures were measured using the indirect tail-cuff method (Kent Scientific, Torrington, CT, USA). Blood samples were collected after a 4 h fast. Plasma concentrations of glucose and total cholesterol were measured using enzymatic methods (Denka Seiken, Tokyo, Japan) [2, 29–37, 39, 40]. The plasma insulin level was measured with an enzyme-linked immunosorbent assay (Ultrasensitive mouse insulin ELISA kit, Morinaga, Yokohama, Japan) [2, 30]. The homeostasis model assessment of insulin resistance (HOMA-IR) was calculated as fasting plasma insulin (pM) × 0.139 (conversion to *μ*U/mL) × fasting plasma glucose (mg/dL)/405 [2, 30].

### 2.12. Assessment of Atherosclerotic Lesions

Before and 4 weeks after the start of infusions, *Apoe*^−/−^ mice were sacrificed by exsanguination (total blood collection) under medetomidine-midazolam-butorphanol anesthesia (0.3 mg/kg, 4.0 mg/kg, and 5.0 mg/kg, respectively, intraperitoneal injection) [2, 29–37, 39, 40]. The whole aorta was washed by perfusion with phosphate-buffered saline and fixed with 4% paraformaldehyde. The aorta was then excised from the aortic root to the abdominal area, and connective and adipose tissues were carefully removed. The entire aorta and cross sections of the aortic root were stained with Oil Red O to assess atherosclerotic lesions [2, 29–37, 39, 40]. Vascular inflammation, monocytes/macrophages, and VSMCs in the aortic atherosclerotic lesions were visualized by staining with antibodies raised against pentraxin-3 (Bioss), MOMA2 (Millipore, Billerica, MA, USA), and *α*-smooth muscle actin (Sigma), respectively [2, 29–37, 39, 40]. These areas of the aortic wall were traced by an investigator blind to the treatment and quantified by image analysis (Adobe Photoshop, San Jose, CA, USA, and NIH ImageJ). In addition, an increased ratio of macrophage contents (*μ*m^2^) : VSMC contents (*μ*m^2^) within the atheromatous plaques was regarded as a marker of plaque instability [2, 30, 32–36].

### 2.13. Statistical Analyses

Data are expressed as the means ± standard error of the mean. The data were compared using the unpaired Student's *t*-test between two groups. Comparison of several groups was performed with one-way analysis of variance followed by Bonferroni's post hoc test. A value of *p* < 0.05 was considered to be statistically significant.

## 3. Results

### 3.1. Expression of *μ*-Opioid Receptor in Human Vascular Cells

**Figure 1:**
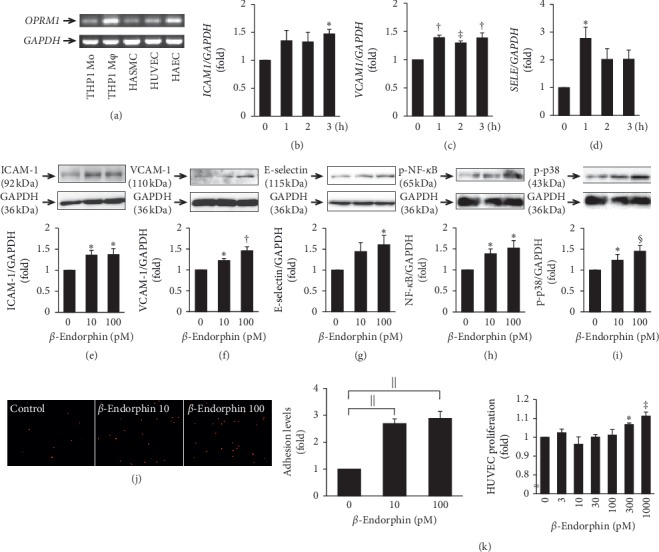
Expression of *μ*-opioid receptor in human vascular cells and effects of *β*-endorphin on the inflammatory response and proliferation of HUVECs. (a) The mRNA expression levels of *OPRM1* (*μ*-opioid receptor gene) in THP-1 monocytes, their derived macrophages, HASMCs, HUVECs, and HAECs were analyzed with RT-PCR. *GAPDH* served as a loading control. Independent experiments were repeated twice to assure reproducibility. (b–d) HUVECs were treated with 100 pM *β*-endorphin for the indicated time (0–3 h). mRNA expression of *ICAM1*, *VCAM1*, *SELE* (E-selectin gene), and *GAPDH* was determined with RT-PCR (*n* = 3). ^*∗*^*p* < 0.05, ^†^*p* < 0.001, ^‡^*p* < 0.005 vs. 0 h (e–i) HUVECs were treated with the indicated concentrations of *β*-endorphin for 24 h and then harvested for immunoblot analyses to evaluate ICAM-1, VCAM-1, and E-selectin protein expression, and NF-*κ*B and p38 phosphorylation (*n* = 4–5). Upper panels show representative immunoblots, and densitometry data after normalization to GAPDH are shown beneath. (j) Confluent HUVECs were incubated in 0.5% FBS-EGM-2 for 16 h and then treated for 4 h with the indicated concentrations of *β*-endorphin. Subsequently, calcein red-orange-labeled THP-1 monocytes were plated on the HUVEC monolayer and incubated for 1 h. After washing, the adherent cells were observed with fluorescence microscopy (*n* = 4). Scale bar = 100 *μ*m. Baseline (1-fold) = 2661.83 ± 128.63 pixels. (k) HUVECs were incubated with the indicated concentrations of *β*-endorphin for 48 h. Proliferation was determined with the WST-8 assay (*n* = 4). (e–k) ^*∗*^*p* < 0.05, ^†^*p* < 0.001, ^‡^*p* < 0.005, ^§^*p* < 0.0005, *p* < 0.0001 vs. 0 pM *β*-endorphin.

First, the gene expression of the *μ*-opioid receptor (*OPRM1*) was investigated in the human vascular cells used in this study. *OPRM1* was expressed at high levels in THP-1 monocyte-derived macrophages and HAECs, and at low levels in THP-1 monocytes, HASMCs, and HUVECs (Figure 1(a)).

### 3.2. Effects of *β*-Endorphin on the Inflammatory Response and Proliferation in Human ECs

We assessed the time-dependent effect of *β*-endorphin on the expression of adhesion molecule genes in HUVECs. *β*-Endorphin (100 pM) significantly enhanced the mRNA expression of *ICAM1*, *VCAM1*, and *SELE* with a maximal effect at 3 h (1.5-fold), 1 h and 3 h (1.4-fold), and 1 h (2.8-fold), respectively (Figures 1(b)–1(d)). Immunoblots also revealed that *β*-endorphin (100 pM) significantly increased the protein expression of ICAM-1 (1.4-fold), VCAM-1 (1.5-fold), and E-selectin (1.6-fold) in association with phosphorylation of NF-*κ*B (1.6-fold) and p38 (1.5-fold) in HUVECs (Figures 1(e)–1(i)).

Exposure of HUVECs to 10 and 100 pM *β*-endorphin for 24 h resulted in 2.7- and 2.9-fold increases in THP-1 monocyte adhesion compared with the untreated control, respectively (Figure 1(j)).

*β*-Endorphin significantly increased the proliferation of HUVECs in a concentration-dependent manner, with a maximal increase of 11% with 100 pM (Figure 1(k)).

### 3.3. Effects of *β*-Endorphin on Foam Cell Formation and Related Protein Expression in Human Macrophages

**Figure 2:**
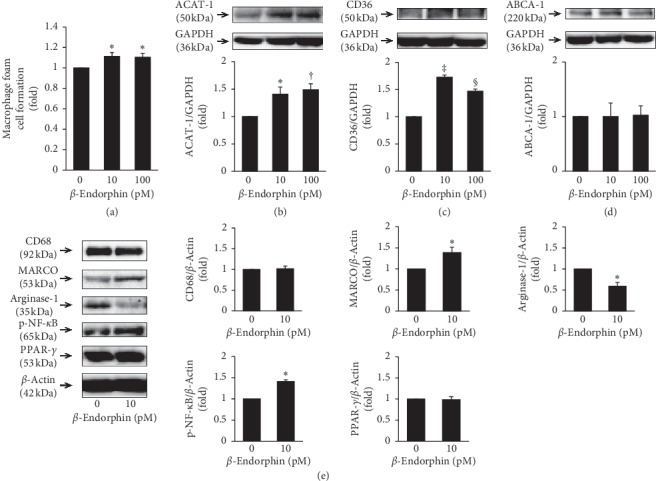
Effects of *β*-endorphin on foam cell formation and related protein expression, and the inflammatory phenotype in THP-1 monocyte-derived macrophages. (a) THP-1 monocytes were incubated for 6 days with the indicated concentrations of *β*-endorphin, followed by a 19 h incubation with 50 *μ*g/mL oxLDL in the presence of 100 *μ*M [^3^H]oleate. Foam cell formation was determined from the intracellular radioactivity of cholesterol-[^3^H]oleate (*n* = 5). Baseline of control = 5.31 ± 0.48 nmol/mg cell protein. (b–d) THP-1 monocytes were incubated for 6 days with the indicated concentrations of *β*-endorphin. THP-1 monocyte-derived macrophages were harvested and used for immunoblot analyses with ABCA1, ACAT-1, CD36, or GAPDH antibodies (*n* = 4). Representative images are shown; the graphs indicate densitometry data following normalization to GAPDH. (e) THP-1 monocytes were incubated for 6 days with or without *β*-endorphin (100 pM) in the presence of TPA (150 ng/mL) to differentiate the cells into macrophages. Cells were harvested and used for immunoblot analyses for CD68 (a macrophage differentiation marker, *n* = 4), MARCO (an M1 macrophage marker, *n* = 3), arginase-1 (an M2 macrophage marker, *n* = 3), PPAR-*γ* (*n* = 3), p-NF-*κ*B (*n* = 3), or GAPDH (*n* = 4). Representative images are shown; the graphs indicate densitometry data following normalization to *β*-actin. (a–e) ^*∗*^*p* < 0.05, ^†^*p* < 0.01, ^‡^*p* < 0.0001, ^§^*p* < 0.005 vs. 0 pM *β*-endorphin.

*β*-Endorphin (10 and 100 pM) significantly increased oxLDL-induced foam cell formation by 1.1-fold in THP-1 monocyte-derived macrophages (Figure 2(a)). *β*-Endorphin (10 and 100 pM) significantly increased ACAT-1 protein expression (1.4-fold and 1.5-fold, respectively) and CD36 expression (1.7-fold and 1.5-fold, respectively), but not ABCA1 expression, in THP-1 monocyte-derived macrophages (Figures 2(b)–2(d)).

### 3.4. Effects of *β*-Endorphin on the Inflammatory Phenotype in Human Macrophages

After 6 days of THP-1 monocyte culture, the differentiation of human monocytes into macrophages was confirmed by increased protein expression of CD68, a macrophage differentiation marker (Figure 2(e)). *β*-Endorphin (10 pM) did not affect the differentiation of monocytes into macrophages. However, *β*-endorphin significantly increased the protein expression of MARCO, an M1 marker, and significantly decreased that of arginase-1, an M2 marker (Figure 2(e)). Likewise, *β*-endorphin significantly increased NF-*κ*B phosphorylation, but not PPAR-*γ* protein expression (Figure 2(e)). These observations indicate that *β*-endorphin shifted the macrophage phenotype to the M1 rather than M2 phenotype, which was associated with NF-*κ*B phosphorylation during monocyte-macrophage differentiation.

### 3.5. Effects of *β*-Endorphin on the Migration, Proliferation, and Signal Transduction in Human VSMCs

**Figure 3:**
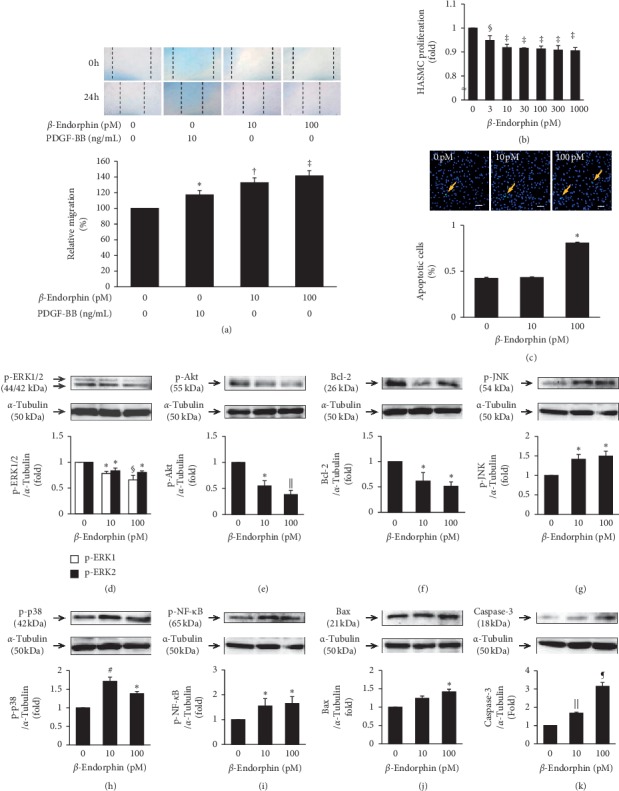
Effects of *β*-endorphin on the migration, proliferation, apoptosis, and signal transduction in HASMCs. (a) Migration was determined with the scratch assay in HASMCs incubated in serum-free SmGM-2 and the indicated concentrations of PDGF-BB or *β*-endorphin. Photos were taken 0 h and 24 h after stimulation. Representative results are shown from eight independent experiments. Graph shows quantification of *β*-endorphin-induced cell migration (*n* = 8). ^*∗*^*p* < 0.05, ^†^*p* < 0.0005, ^‡^*p* < 0.0001 vs. 0 pM *β*-endorphin + 0 ng/mL PDGF-BB. (b) Proliferation of HASMCs was determined with the WST-8 assay following a 48 h incubation in 5% FBS-SmGM-2 with the indicated concentrations of *β*-endorphin (*n* = 4). (c) HASMCs treated with *β*-endorphin for 48 h were stained to detect apoptotic cells (green) using the TUNEL method. Nuclei were stained using DAPI (blue). The graph indicates the percentage of apoptotic cells in three independent experiments. Scale bar = 100 *μ*m. (d–k). HASMCs were incubated in 5% FBS-SmGM-2 with *β*-endorphin (0, 10, and 100 pM) for 24 h. The effects of *β*-endorphin on intracellular signals were assessed with immunoblot analyses. Densitometric data for each molecule after normalization to *α*-tubulin (p-ERK1/2, *n* = 3; p-Akt, *n* = 4; Bcl-2, *n* = 4; p-JNK, *n* = 4; p-p38, *n* = 3; p-NF-*κ*B, *n* = 4; Bax, *n* = 4; cleaved caspase-3, *n* = 3). (b–k) ^*∗*^*p* < 0.05, ^§^*p* < 0.005, *p* < 0.01, ^#^*p* < 0.001, *p* < 0.0001 vs. 0 pM *β*-endorphin.

We performed a scratch assay to determine *β*-endorphin-mediated cell motility. Treatment with *β*-endorphin (10 and 100 pM) significantly increased HASMC migration more than PDGF-BB (10 ng/mL) (Figure 3(a)). Treatment with *β*-endorphin for 48 h significantly decreased HASMC proliferation in a concentration-dependent manner (Figure 3(b)). Moreover, *β*-endorphin (100 pM) significantly induced apoptosis in HASMCs as seen with the TUNEL assay (Figure 3(c)). *β*-Endorphin (10 and 100 pM) significantly decreased phosphorylation of ERK1/2 and Akt, and Bcl-2 protein expression. In contrast, *β*-endorphin significantly increased phosphorylation of JNK, p38, and NF-*κ*B, Bax, and cleaved caspase-3 expression (Figures 3(d)–3(j)).

### 3.6. Effects of *β*-Endorphin on Atherosclerotic Lesion Development in Apoe^−/−^ Mice

**Figure 4:**
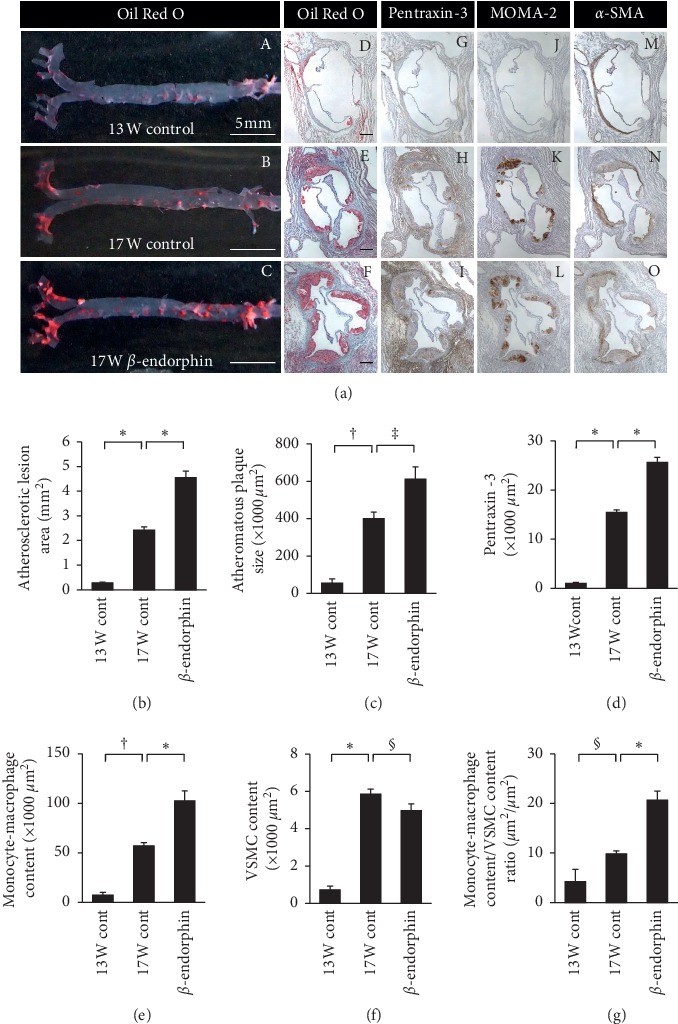
Effects of *β*-endorphin on atherosclerotic lesion development in *Apoe*^−/−^ mice. Of 21 *Apoe*^−/−^ mice at 13 weeks of age, five were sacrificed before infusion, and eight each were infused with saline or *β*-endorphin (5 *μ*g/kg/h) using osmotic minipumps for 4 weeks. (a) The aortic surface was stained with Oil Red O (a–c). Cross sections of the aortic root were stained with Oil Red O (d–f), with immunostaining for pentraxin-3 (g–i), MOMA-2 (j–l), or *α*-smooth muscle actin (m–o). Scale bar = 200 *μ*m. (b–g) Statistical comparisons of the atherosclerotic lesion area, atheromatous plaque size, pentraxin-3-positive area, monocyte/macrophage infiltration, and VSMC contents, and the ratio of monocyte-macrophage contents : VSMC contents within atheromatous plaques among three groups. Data are presented as the means ± standard error of the mean. ^*∗*^*p* < 0.0001, ^†^*p* < 0.0005, ^‡^*p* < 0.005, ^§^*p* < 0.05.

In *Apoe*^−/−^ mice, the aortic atherosclerotic lesion area and atheromatous plaque size accompanied by the intraplaque pentraxin-3-positive area and monocyte/macrophage and VSMC contents as well as plasma total cholesterol concentration were significantly increased at 17 weeks of age compared with 13 weeks of age by 8.8-fold, 7.2-fold, 15.2-fold, 7.9-fold, and 8.1-fold, respectively (Figure 4(a) (A, B, D, G, H, J, K, M, and N) and 4(b)–4(f) and Table 1). Infusion of *β*-endorphin (5 *μ*g/kg/h) significantly enhanced the aortic atherosclerotic lesion area and atheromatous plaque size by 1.9-fold and 1.5-fold, respectively (Figure 4(a) (B, C, E, and F) and 4(b) and 4(c)), with significant increases in the pentraxin-3-positive area and monocyte/macrophage infiltration by 1.7-fold and 2.1-fold, respectively, and a significant decrease in the VSMC content (Figure 4(a) (H, I, K, L, N, and O) and 4(d)–4(f)). In addition, the ratio of monocyte/macrophage contents : VSMC contents within atheromatous plaques was significantly increased by *β*-endorphin by 2.1-fold (Figure 4(g)). We found no significant differences in body weight; food intake; systolic and diastolic blood pressures; plasma levels of total cholesterol, triglycerides, free fatty acids, glucose, and insulin; or HOMA-IR between the two groups of 17-week-old *Apoe*^−/−^ mice (Table 1).

## 4. Discussion

The present study provides the first evidence that *β*-endorphin stimulates atherosclerosis by increasing the inflammation in ECs and macrophages, mediating oxLDL-induced foam cell formation in association with CD36 and ACAT-1 upregulation, and inducing migration and apoptosis in VSMCs. Moreover, *β*-endorphin accelerates vascular inflammation and the development and instability of atherosclerotic plaques in *Apoe*^−/−^ mice.

We discuss the reason why *β*-endorphin increases HUVEC proliferation but decreases HASMC proliferation. A previous study has shown that *β*-endorphin stimulates the migration and proliferation of HUVECs, leading to angiogenesis [41]. This report is consistent with our results. We also showed that *β*-endorphin increased the migration HASMCs via stimulation of JNK phosphorylation. However, in these cells, the peptide decreased proliferation and increased apoptosis via suppression of ERK1/2 and Akt phosphorylation and Bcl-2 expression, and via stimulation of p38, Bax, and caspase-3 expression. Parra et al. have reported that *μ*-opioid receptor agonists show biphasic action on vasocontraction, which is suppressed at low concentrations but stimulated at high concentrations [42]. In the present study, *β*-endorphin reduced the proliferation of VSMCs in vitro and in vivo and thus contributed to the instability of atheromatous plaques.

Several studies have shown that plasma *β*-endorphin levels increase with psychological stress and exercise stress in patients with CAD [43, 44]. Plasma *β*-endorphin levels at onset are increased in patients with acute myocardial infarction with chest pain compared to those without chest pain [45]. In patients with symptomatic myocardial ischemia, plasma *β*-endorphin levels decrease after percutaneous transluminal coronary angioplasty [46]. Plasma *β*-endorphin levels are increased in patients with essential hypertension and heart failure [28, 47]. Cozzolino et al. have shown that a short-term infusion of *β*-endorphin improves cardiac function and systemic vascular resistance in patients with heart failure [48]. These findings suggest that *β*-endorphin may play a key role in cardioprotection against pain, stress, and pressure overload. However, the present study reveals that long-term infusion of *β*-endorphin accelerates the progression and instability of atheromatous plaques. Further studies are needed to demonstrate the stimulatory effect of *β*-endorphin on plaque rupture in aged *Apoe*^−/−^mice.

The physiological relevance of *β*-endorphin concentrations used in the present in vitro and in vivo experiments warrants further discussion. First, the concentrations of *β*-endorphin (10–100 pM) that were needed to influence multiple responses by HUVECs, THP-1 monocyte-derived macrophages, and HASMCs were almost equivalent to the average plasma concentration of *β*-endorphin in patients with CAD (10–75 pM) [27, 49]. *β*-Endorphin affects HASMCs at a maximum concentration of 1000 pM, which is ∼100-fold higher than the plasma concentration. In the vascular wall, ECs and macrophages generate high amounts of *β*-endorphin in an autocrine/paracrine manner [15]. In a previous study, local levels of angiotensin II were increased by ∼100-fold [50]. Therefore, the observation that local levels of *β*-endorphin were increased in the microenvironment of the cells that secrete the peptide to a similar degree as other vasoactive agents is not surprising.

The present study has some limitations. Future experiments with overexpression of *β*-endorphin, knockout of the *μ*-opioid receptor, and administration of a selective *μ*-opioid receptor antagonist, naloxonazine dihydrochloride, in *Apoe*^−/−^ mice may strengthen our observation of the stimulatory effects of *β*-endorphin on atherosclerosis. Likewise, experiments using *β*-endorphin with naloxonazine dihydrochloride may be required in all in vitro experiments in future studies.

## 5. Conclusions

The results of the present study indicated that *β*-endorphin accelerates atherosclerosis by enhancing inflammatory responses and monocyte adhesion in ECs, the inflammatory phenotype and foam cell formation in macrophages, and migration of VSMCs. In addition, *β*-endorphin may contribute to plaque instability by increasing vascular inflammation and the intraplaque macrophage : VSMC ratio. Therefore, *μ*-opioid receptor antagonists may serve as novel potential therapeutic agents for atherosclerosis and related diseases.

## Figures and Tables

**Table 1:** Characteristics and laboratory data of *Apoe*^−/−^ mice.

Parameter	13 weeks old	17 weeks old	
		Control	*β*-Endorphin
N	5	8	8
Body weight (g)	25.5 ± 0.9	29.3 ± 0.5^*∗*^	29.9 ± 0.4^†^
Food intake (g/day)	NE	3.7 ± 0.2	3.7 ± 0.4
Systolic blood pressure (mmHg)	91.3 ± 0.6	91.4 ± 0.8	92.1 ± 1.2
Diastolic blood pressure (mmHg)	73.4 ± 0.8	72.5 ± 0.6	72.2 ± 0.6
Total cholesterol (mg/dL)	495.0 ± 64.9	1784.3 ± 62.4^‡^	1706.1 ± 75.0^‡^
Triglyceride (mg/dL)	202.2 ± 8.4	198.3 ± 12.8	212.1 ± 14.7
Free fatty acid (mEq/L)	1.3 ± 0.3	1.7 ± 0.1	1.5 ± 0.1
Glucose (mg/dL)	266.5 ± 32.9	305.2 ± 14.4	294.3 ± 18.3
Insulin (pM)	22.4 ± 4.1	23.7 ± 4.4	31.5 ± 3.4
HOMA-IR	2.3 ± 0.4	2.5 ± 0.5	3.1 ± 0.3

Measurements of body weight, food intake, systolic and diastolic blood pressures, and fasting plasma parameters were performed before (13 weeks old control) and after 4-week infusion (17 weeks old) of saline or *β*-endorphin in *Apoe*^−/−^ mice. ^*∗*^*p* < 0.005, ^†^*p* < 0.001, ^‡^*p* < 0.0001 vs. 13 weeks old control. NE = not examined.

## Data Availability

All data used to support the findings of this study are available from the corresponding author upon request.
